# Towards a safer probiotic: *clbD* deletion attenuates EcN-induced DNA damage responses and epithelial inflammatory transcriptional responses

**DOI:** 10.3389/fcimb.2026.1812973

**Published:** 2026-08-06

**Authors:** Wei-Ming Chen, Ming-Yang Sun, Ji-Yuan Ye, De-Cheng Tan, Zi-Yi Zhang, Jing-Jing Liu

**Affiliations:** 1 Department of Gastroenterology, The National Key Clinical Specialty, Zhongshan Hospital of Xiamen University, School of Medicine, Xiamen University, Fujian, China; 2 Clinical Research Center for Gut Microbiota and Digestive Diseases of Fujian Province, Xiamen Key Laboratory of Intestinal Microbiome and Human Health, Xiamen, Fujian, China; 3 Department of Digestive Disease, Institute for Microbial Ecology, School of Medicine, Xiamen University, Xiamen, Fujian, China

**Keywords:** chromosomal complementation, *clbD*, colibactin, DNA damage response, *Escherichia coli* Nissle 1917, inflammatory transcription, *pks* genomic island, probiotic safety

## Abstract

*Escherichia coli* Nissle 1917 (EcN) is widely used as a probiotic and engineering chassis, but its carriage of the *pks* genomic island raises safety concerns related to the genotoxin colibactin. Here, we constructed a *clbD* deletion mutant (EcNΔ*clbD*) and a chromosomally complemented strain (EcNΔ*clbD*::*clbD*) using a CRISPR/Cas9-based genome-editing strategy. Locus-specific PCR confirmed the expected *clbD* deletion and restoration patterns. DNA damage responses were evaluated by γH2AX immunofluorescence and alkaline comet assay after bacterial co-culture. Compared with EcN, EcNΔ*clbD* markedly reduced γH2AX positivity and comet-positive cells, whereas *clbD* complementation restored both phenotypes to levels comparable to EcN. In NCM460 colonic epithelial cells, EcNΔ*clbD* induced lower mRNA expression of pro-inflammatory cytokines and TLR4–MYD88–NFKB1-related transcripts than EcN. Additional CCK-8 and cell-associated bacterial load assays showed no significant differences between EcN- and EcNΔ*clbD*-treated groups, suggesting that the reduced inflammatory transcription was not primarily attributable to altered host cell CCK-8 readout or bacterial exposure. qPCR analysis of neighboring *pks* genes showed detectable local transcriptional changes after *clbD* deletion that were restored toward the parental EcN pattern by complementation. Transmission electron microscopy revealed no detectable morphological or ultrastructural disruption after *clbD* deletion. Exploratory untargeted metabolomics suggested a metabolic shift associated with *clbD* deletion, while gastrointestinal stress assays revealed condition-dependent fitness effects without a generalized growth defect in LB medium. Together, these findings indicate that *clbD* deletion attenuates EcN-induced DNA damage responses and epithelial inflammatory transcription *in vitro*. EcNΔ*clbD* may represent a candidate safety-optimized EcN chassis requiring further validation in additional epithelial models and *in vivo* systems.

## Introduction

*Escherichia coli* Nissle 1917 (EcN) has been used for decades as a probiotic and, more recently, has attracted increasing interest as a microbial chassis for engineered live biotherapeutics and customizable drug-delivery systems because of its genetic tractability and ability to persist transiently in the gut (Kruis et al., 2004; Lynch et al., 2022; Chen et al., 2023a; Yu et al., 2023; Murali and Mansell, 2024). At the same time, the translation of engineered probiotics toward clinical use has sharpened expectations regarding strain identity, genetic stability, manufacturing controls, and risk assessment under live biotherapeutic product regulatory frameworks (Cordaillat-Simmons et al., 2020; Rouanet et al., 2020; Tseng et al., 2025).

A key safety concern for EcN is that it harbors the *pks* genomic island, which encodes the biosynthetic machinery for the genotoxin colibactin (Nougayrède et al., 2006; Chagneau et al., 2022; Tang et al., 2022). Colibactin-producing *pks*-positive *Escherichia coli* can induce DNA damage responses in host cells and has been linked to a distinct mutational signature detected in a subset of human colorectal cancers, supporting the possibility that prior exposure to *pks*-positive bacteria may contribute to carcinogenesis (Nougayrède et al., 2006; Pleguezuelos-Manzano et al., 2020; Jans et al., 2024; Ouyang and Puschhof, 2024; Rosendahl Huber et al., 2024; Zhang and Sun, 2024; Jans and Vereecke, 2025). Importantly, EcN itself has been shown to produce colibactin and to induce mutagenic DNA damage *in vitro* and *in vivo*, raising renewed questions about how to preserve probiotic utility while reducing genotoxicity-associated risk (Massip et al., 2019; Nougayrède et al., 2021).

Safety-by-design strategies include genetically disabling the colibactin pathway while monitoring whether probiotic-relevant bacterial characteristics are retained (Massip et al., 2019; Kalantari et al., 2023). Complementary chemical biology approaches, such as pharmacological targeting of the colibactin-associated genotoxic pathway, also provide mechanistic tools and reinforce the central role of the *pks* pathway in colibactin-associated genotoxicity (Volpe et al., 2023). However, attributing host-response changes to a specific *pks* gene deletion requires appropriate genetic controls, including chromosomal complementation, as well as orthogonal assays for DNA damage responses.

Here, we engineered an EcN *clbD* deletion mutant (EcNΔ*clbD*) and a chromosomally complemented strain (EcNΔ*clbD*::*clbD*) to evaluate the contribution of *clbD* to EcN-induced host responses. We performed a multi-layer characterization spanning DNA damage response assessment by γH2AX immunofluorescence and alkaline comet assay, inflammatory transcriptional profiling in intestinal epithelial cells, bacterial morphology, local *pks* gene transcription, untargeted metabolomics, and gastrointestinal stress tolerance. This work aims to evaluate EcNΔ*clbD* as a candidate safety-optimized EcN chassis while explicitly considering the limitations of short-term *in vitro* assays and the possibility of context-dependent fitness trade-offs under gut-relevant stressors.

## Materials and methods

### Bacterial strains, plasmids, and growth conditions

*Escherichia coli* Nissle 1917 (EcN) was obtained from the commercial probiotic product Mutaflor^®^ and used as the parental strain. The *clbD* deletion mutant, EcNΔ*clbD*, was generated from EcN using a CRISPR/Cas9-based genome-editing strategy. The chromosomally complemented strain, EcNΔ*clbD*::*clbD*, was generated by restoration of *clbD* in EcNΔ*clbD* at the native *clbD* locus. The CRISPR/Cas9 editing plasmids pCas (Addgene plasmid #62225) and pTargetF (Addgene plasmid #62226) were obtained from Addgene and used as described previously (Jiang et al., 2015). The original pTargetF plasmid was used as the parental editing backbone. After assembly of the corresponding sgRNA cassette and donor DNA fragments, the resulting editing plasmids were designated pTargetT-Δ*clbD* and pTargetT-*clbD* for *clbD* deletion and chromosomal complementation, respectively.

Unless otherwise specified, bacteria were cultured in Luria–Bertani (LB) broth containing 1% tryptone, 0.5% yeast extract, and 1% NaCl at 37 °C with shaking at 200 rpm. For routine propagation, overnight cultures were grown for approximately 12 h and used for downstream experiments. When required, strains were plated on LB agar and incubated at 37 °C.

### Construction of EcNΔ*clbD* and EcNΔ*clbD*::*clbD*

The original pTargetF plasmid was used as the parental pTarget-series backbone for constructing editing plasmids for *clbD* deletion and chromosomal complementation. Two 20-nt spacer sequences targeting the *clbD* locus were designed using the CHOPCHOP online gRNA design tool: one for *clbD* deletion and the other for chromosomal complementation. For *clbD* deletion, donor DNA fragments containing the upstream and downstream homology arms flanking the *clbD* deletion region, together with the corresponding gRNA expression cassette, were assembled into the linearized pTarget backbone using the ClonExpress II One Step Cloning Kit (Vazyme), generating pTargetT-Δ*clbD*. For chromosomal complementation, donor DNA containing the *clbD* sequence flanked by homologous regions, together with the corresponding gRNA expression cassette, was assembled into the linearized pTarget backbone using the same strategy, generating pTargetT-*clbD*.

For genome editing, EcN or EcNΔ*clbD* carrying pCas was transformed with the corresponding pTargetT plasmid. Candidate colonies were screened by locus-specific PCR using primers flanking the *clbD* region. The expected PCR amplicon sizes were 1561 bp for EcN, 691 bp for EcNΔ*clbD*, and 1561 bp for EcNΔ*clbD*::*clbD*. Positive clones were used for downstream phenotypic assays. All oligonucleotides used for construction and PCR validation of the *clbD* deletion and complementation strains are provided in **Supplementary Table 1**.

### Mammalian cell culture

Human normal colonic epithelial NCM460 cells were maintained at 37 °C in a humidified incubator with 5% CO2. The NCM460 human colonic epithelial cell line was obtained from SBC (Shanghai) Biotechnology Co., Ltd. (Saibaikang, Shanghai, China; catalog no. iCell-h373) and authenticated by short tandem repeat (STR) profiling. NCM460 cells were cultured in RPMI-1640 supplemented with 10% fetal bovine serum (FBS) and 1% penicillin–streptomycin (P/S).

HeLa human cervical cancer cells were used as an additional DNA damage response validation model, consistent with previous studies assessing *pks*-positive *E. coli*- or EcN-induced genotoxicity. HeLa cells were obtained from SBC (Shanghai) Biotechnology Co., Ltd. (Saibaikang, Shanghai, China; catalog no. iCell-h088) and authenticated by STR profiling. HeLa cells were cultured in DMEM supplemented with 10% FBS and 1% P/S.

Cells were routinely passaged at approximately 80–90% confluency using trypsin–EDTA and seeded at appropriate densities depending on the downstream assay. For bacterial co-culture experiments, cells were seeded one day before infection to reach approximately 70–80% confluency on the day of co-culture, and antibiotics were omitted during bacterial exposure.

### Bacterial–epithelial co-culture

For DNA damage response assays, HeLa cells and NCM460 cells were co-cultured with EcN, EcNΔ*clbD*, or EcNΔ*clbD*::*clbD* at a multiplicity of infection (MOI) of 10 for 4 h. HeLa cells were included as an additional DNA damage response validation model, whereas NCM460 cells were used to evaluate DNA damage responses and inflammatory transcriptional responses in a non-tumorigenic colonic epithelial context. For inflammatory transcriptional analysis, NCM460 cells were treated with PBS, EcN, or EcNΔ*clbD* under the same co-culture conditions. Bacterial inocula were prepared from overnight cultures grown in LB at 37 °C with shaking. Bacteria were washed twice with sterile PBS, and the inoculum was adjusted to the desired MOI based on OD600. Co-culture was performed in antibiotic-free complete medium. RPMI-1640 supplemented with 10% FBS was used for NCM460 cells, and DMEM supplemented with 10% FBS was used for HeLa cells. After 4 h, cells were washed with PBS and processed immediately for downstream assays, including fixation for γH2AX immunofluorescence staining, alkaline comet assay, RNA extraction, CCK-8 assay, or cell-associated bacterial load determination.

### γH2AX immunofluorescence staining

After bacterial–epithelial co-culture, cells were washed with PBS, and DNA damage-associated signaling was assessed by γH2AX immunofluorescence using a commercial DNA Damage Assay Kit (γH2AX Immunofluorescence) (Beyotime, Cat. No. C2035S) according to the manufacturer’s instructions. γH2AX, corresponding to H2AX phosphorylated at Ser139, is a widely used marker of DNA double-strand break-associated signaling (Rogakou et al., 1998). Briefly, cells were fixed, blocked, and incubated with the kit-provided anti-γH2AX primary antibody, followed by the supplied Alexa Fluor 488-conjugated anti-rabbit secondary antibody. Nuclei were counterstained with the DAPI solution included in the kit, and samples were mounted with the supplied antifade mounting medium. Images were acquired using an inverted fluorescence microscope under identical acquisition settings across groups. γH2AX positivity was quantified as the percentage of γH2AX-positive cells among total DAPI-stained nuclei using predefined criteria.

### Alkaline comet assay

DNA strand break-related migration was further assessed using an alkaline comet assay after bacterial–epithelial co-culture. After exposure to PBS, EcN, EcNΔ*clbD*, or EcNΔ*clbD*::*clbD*, cells were washed with PBS and processed using a comet assay kit (Beyotime, Cat. No. C2041M) according to the manufacturer’s instructions. Briefly, cells were embedded in low-melting-point agarose on comet slides, lysed under alkaline conditions, subjected to alkaline unwinding and electrophoresis, and then neutralized and stained with DNA dye. Images were acquired using a fluorescence microscope under identical imaging settings across groups. Comet-positive cells were quantified based on visible comet tail formation. For each group, 100 cells were analyzed per biological replicate, and three biological replicates were included.

### RNA extraction and qPCR analysis of epithelial inflammatory transcription

After bacterial co-culture, total RNA was extracted from NCM460 cells using TRIzol reagent according to the manufacturer’s instructions. The acid guanidinium thiocyanate–phenol–chloroform principle underlying TRIzol-based extraction was originally described by Chomczynski and Sacchi (1987). Residual genomic DNA was removed, and cDNA was synthesized using HiScript II Q Select RT SuperMix for qPCR (+gDNA wiper) (Vazyme, Cat. No. R233-01) following the supplier’s protocol. Quantitative PCR was performed on a Bio-Rad CFX96 real-time PCR system using the 2× One Step SYBR Green Master Mix from the UniPeak U+ One Step RT-qPCR SYBR Green Kit (Vazyme, Cat. No. Q226-01). Target genes included pro-inflammatory cytokines and genes related to the TLR4–MYD88–NFKB1 axis. GAPDH served as the internal control. Relative expression was calculated using the 2−ΔΔCt method (Livak and Schmittgen, 2001). Primer sequences are provided in **Supplementary Table 2**. Each condition included three biological replicates, and each sample was run in technical triplicate.

### qPCR analysis of local *pks* gene transcription

To evaluate potential local transcriptional effects of *clbD* deletion within the *pks* island, bacterial RNA was extracted from EcN, EcNΔ*clbD*, and EcNΔ*clbD*::*clbD* cultures grown under comparable conditions. Total bacterial RNA was extracted using TRIzol reagent according to the manufacturer’s instructions. Residual genomic DNA was removed, and cDNA was synthesized using HiScript II Q Select RT SuperMix for qPCR (+gDNA wiper) (Vazyme, Cat. No. R233-01). Quantitative PCR was performed on a Bio-Rad CFX96 real-time PCR system using the 2× One Step SYBR Green Master Mix from the UniPeak U+ One Step RT-qPCR SYBR Green Kit (Vazyme, Cat. No. Q226-01). The expression of *clbC*, *clbD*, and *clbE* was quantified. Relative expression was calculated using the 2−ΔΔCt method, with *rpoB* used as the internal control. Primer sequences are provided in **Supplementary Table 2**.

### CCK-8 assay after bacterial co-culture

To assess whether differences in inflammatory transcription could be explained by differential host cell metabolic activity between bacterial exposure conditions, a CCK-8 assay was performed after bacterial co-culture. NCM460 cells were co-cultured with EcN or EcNΔ*clbD* as described above. After bacterial exposure, cells were washed twice with PBS to remove non-adherent bacteria and then incubated in antibiotic-containing medium before CCK-8 measurement. CCK-8 reagent (Beyotime, Cat. No. C0039) was added according to the manufacturer’s instructions, and absorbance was measured at 450 nm using a microplate reader. Results were interpreted as a host cell metabolic activity readout rather than a comprehensive cell death assay.

### Cell-associated bacterial load assay

To determine whether EcN and EcNΔ*clbD* differed in effective cell-associated bacterial burden after co-culture, cell-associated bacterial loads were quantified by CFU enumeration. After bacterial co-culture, NCM460 cells were washed twice with sterile PBS to remove non-adherent bacteria. Cells were then lysed under sterile conditions using sterile PBS containing 0.1% Triton X-100, serially diluted in sterile PBS, and plated on LB agar. Plates were incubated at 37 °C, and colonies were counted to calculate cell-associated bacterial loads. Results were expressed as CFU/mL.

### Transmission electron microscopy

Transmission electron microscopy sample preparation and imaging were performed by the Xiamen University Instrument Sharing Platform using standard protocols. EcN and EcNΔ*clbD* were harvested and processed for ultrathin sectioning at approximately 70 nm, followed by contrasting with uranyl acetate and lead citrate. Images were acquired using a Tecnai Spirit transmission electron microscope operated at 120 kV. Three independent sample preparations were performed for each strain, and representative images are shown.

### Untargeted metabolomics

Untargeted metabolomics was performed on bacterial pellets from EcN and EcNΔ*clbD*, with three biological replicates per group, by Wuhan Benagen Tech Solutions Co., Ltd. (Wuhan, China). Metabolites were extracted using a −40 °C pre-chilled internal-standard-containing solvent consisting of methanol:acetonitrile:water at a ratio of 2:2:1, followed by homogenization or sonication, centrifugation, and LC–MS/MS analysis of the supernatants, consistent with widely used LC–MS global metabolite profiling workflows (Want et al., 2010). LC–MS/MS was conducted on a Vanquish UHPLC system coupled to an Orbitrap Exploris 120 high-resolution mass spectrometer (Thermo Fisher Scientific) using a BEH Amide HILIC column (Waters; 2.1 mm × 50 mm, 1.7 μm) in both positive and negative ion modes.

Raw data were converted to mzXML format using ProteoWizard (Chambers et al., 2012) and processed for peak detection, alignment, feature extraction, and metabolite annotation against an in-house database. Features were filtered to reduce noise and missing-value bias. Outlier feature filtering was performed based on the interquartile range, and missing-value filtering retained features with no more than 50% missing values within a single group or across all groups. Missing values were imputed using one-half of the minimum value. Data were normalized using total ion current normalization before downstream analysis. Only MS2-supported metabolite annotations were retained for biological interpretation.

Principal component analysis was used as an unsupervised overview of sample distribution. OPLS-DA was performed using SIMCA software (version 18.0.1; Sartorius Stedim Data Analytics AB, Umea, Sweden) after logarithmic transformation and UV scaling. Seven-fold cross-validation was used to evaluate model quality, and 200 permutation tests were performed to assess potential overfitting. Differential metabolite screening combined OPLS-DA-derived variable importance in projection values (Bylesjö et al., 2007) with Student’s t-test P values. Candidate metabolites were defined using VIP > 1 and P < 0.05 and were used for overview visualization and pathway enrichment analysis. For consistency with the Results and figures, fold changes were presented as EcNΔ*clbD* relative to EcN. Given the exploratory nature of this untargeted metabolomics analysis, the limited sample size, and the absence of multiple-testing correction, these results were interpreted as hypothesis-generating only. KEGG pathway enrichment was performed for candidate metabolites with KEGG compound identifiers using Fisher’s exact test (Ogata et al., 1999).

### Gastrointestinal stress tolerance assays

Gastrointestinal stress tolerance assays were conducted to evaluate bacterial survival under acid, bile salt, and digestive enzyme challenges, following commonly used *in vitro* stress-testing principles for probiotics (Wendel, 2021). EcN and EcNΔ*clbD* were grown to a comparable growth phase, collected by centrifugation, washed with sterile PBS, and resuspended to a standardized inoculum of approximately 10^8 CFU/mL. For acid tolerance, bacterial suspensions were transferred into 0.1 M glycine–HCl buffer containing pepsin at 0.3% (w/v), adjusted to pH 2.0 or pH 3.0, and incubated aerobically at 37 °C for 3 h, with pH 6.2 buffer used as the control condition. For bile tolerance, bacterial suspensions were incubated aerobically at 37 °C for 12 h in Oxgall at final concentrations of 0.3%, 0.5%, or 1.0% (w/v), a commonly used bile source in probiotic bile-tolerance testing (Hu et al., 2018). For enzyme tolerance, bacterial suspensions were incubated aerobically at 37 °C for 3 h with α-amylase, proteinase K, or lipase, each at a final concentration of 1 mg/mL, using enzyme-free PBS at pH 7.0 as the control condition.

After each treatment, viable bacteria were enumerated by serial dilution and plating on LB agar to determine CFU. Survival was calculated as 100 × (CFU after treatment/CFU at baseline). At least three independent biological replicates were performed per condition.

### Baseline bacterial growth assay

To determine whether *clbD* deletion caused a generalized growth defect under standard culture conditions, baseline growth curves were generated for EcN and EcNΔ*clbD* in LB medium. Overnight bacterial cultures were diluted into fresh LB broth to the same starting OD600. Cultures were incubated at 37 °C with shaking at 200 rpm, and OD600 was measured at regular intervals using a spectrophotometer or microplate reader. Growth curves were generated from at least three independent biological replicates.

### Ethics statement

This study did not involve human participants or animals. All experiments were performed using established cell lines obtained from a commercial supplier; therefore, ethical approval and informed consent were not required.

### Statistical analysis

Statistical analyses were performed using GraphPad Prism. Data are presented as independent biological replicates and shown as mean ± SEM unless otherwise specified. For comparisons among three or more groups, one-way analysis of variance followed by Tukey’s multiple-comparisons test was applied. For direct two-group comparisons, including CCK-8 absorbance and cell-associated bacterial loads between EcN and EcNΔ*clbD*, unpaired two-tailed Student’s t-tests were used. For gastrointestinal stress tolerance assays, data were analyzed using two-way ANOVA followed by Šídák’s multiple-comparisons test within each stress category. For baseline bacterial growth curves, repeated measurements over time were analyzed by two-way ANOVA. For comet assay quantification, 100 cells were analyzed per biological replicate, with three biological replicates per group. All tests were two-sided, and P < 0.05 was considered statistically significant.

## Results

### Construction and validation of *clbD*-deleted and *clbD*-complemented EcN strains

**Figure 1:**
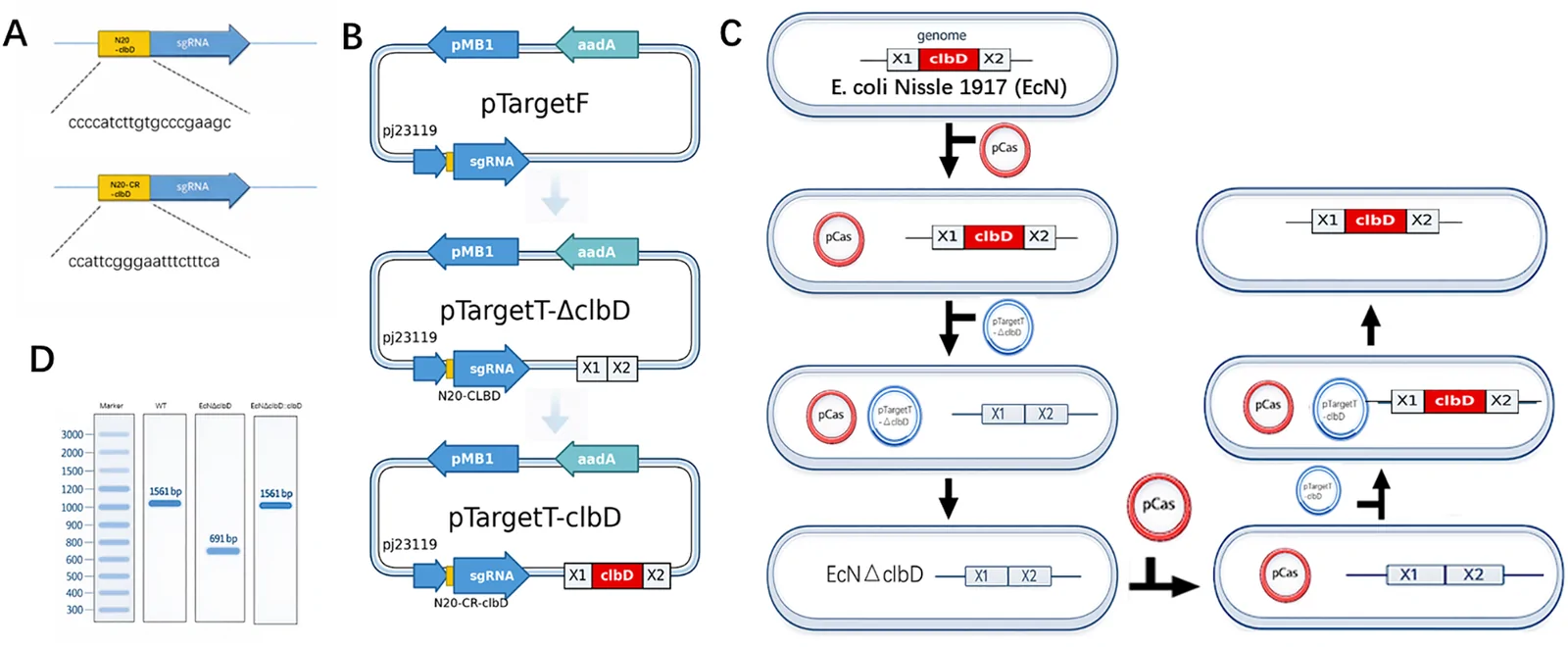
Construction and validation of *clbD* deletion and complemented EcN strains. **(A)** Schematic design of sgRNAs targeting the *clbD* locus for gene deletion and seamless chromosomal complementation. The corresponding protospacer sequences are shown below each sgRNA schematic. **(B)** Construction of pTarget-based editing plasmids. The original pTargetF backbone was used as the parental editing vector. After insertion of the corresponding sgRNA cassette and donor fragments, the resulting editing plasmids were designated pTargetT-Δ*clbD* for *clbD* deletion and pTargetT-*clbD* for chromosomal *clbD* complementation. **(C)** CRISPR/Cas9-mediated genome editing workflow for generating EcNΔ*clbD* and the complemented strain EcNΔ*clbD*::*clbD*. EcN was first edited to delete *clbD* through Cas9 cleavage and homology-directed repair. The EcNΔ*clbD* strain was subsequently subjected to seamless chromosomal complementation to restore *clbD* at the native locus. **(D)** Locus-specific PCR verification of EcN, EcNΔ*clbD*, and EcNΔ*clbD*::*clbD*. EcN produced a 1561-bp amplicon, EcNΔ*clbD* produced a 691-bp amplicon, and the complemented strain restored the 1561-bp amplicon, consistent with successful *clbD* deletion and chromosomal complementation.

To generate a *clbD*-deleted EcN strain and to further verify the causal contribution of *clbD*, we constructed both a *clbD* deletion mutant and a chromosomally complemented strain using a CRISPR/Cas9-based genome-editing strategy. First, sgRNAs targeting the *clbD* locus were designed for gene deletion and seamless chromosomal complementation (**Figure 1A**). The original pTargetF backbone was used to construct the pTarget-based editing plasmids. After insertion of the corresponding sgRNA cassette and donor DNA fragments, the resulting plasmids were designated pTargetT-Δ*clbD* and pTargetT-*clbD*, respectively (**Figure 1B**).

EcN was first edited with pCas and pTargetT-Δ*clbD*. Cas9-mediated cleavage followed by homology-directed repair generated an 870-bp deletion at the *clbD* locus, yielding EcNΔ*clbD*. To restore *clbD*, EcNΔ*clbD* was subsequently subjected to a second round of genome editing using pTargetT-*clbD*, in which the *clbD* sequence was reintroduced at the native chromosomal locus to generate the complemented strain EcNΔ*clbD*::*clbD* (**Figure 1C**). Locus-specific PCR confirmed the expected amplicon size shift among the three strains: EcN produced a 1561-bp band, EcNΔ*clbD* produced a 691-bp band, and EcNΔ*clbD*::*clbD* restored the 1561-bp band (**Figure 1D**; **Supplementary Figure 1**). These results supported the successful construction of both the *clbD* deletion mutant and the chromosomally complemented strain for subsequent functional analyses.

To assess whether *clbD* deletion affected local transcription within the *pks* island, we measured the mRNA expression of *clbD* and its neighboring genes *clbC* and *clbE* by qPCR. As expected, *clbD* expression was almost undetectable in EcNΔ*clbD* and was restored in the complemented strain EcNΔ*clbD*::*clbD*. Compared with EcN, EcNΔ*clbD* showed increased mRNA expression of the adjacent genes *clbC* and *clbE*. Notably, chromosomal complementation of *clbD* reduced *clbC* and *clbE* expression to levels comparable to those observed in EcN. These results indicate that *clbD* deletion was associated with detectable local transcriptional changes in neighboring *pks* genes, whereas *clbD* complementation restored the local transcriptional profile toward the parental EcN pattern (**Supplementary Figure 2**).

### *clbD* deletion attenuates EcN-induced DNA damage responses, whereas chromosomal complementation restores DNA damage responses

**Figure 2:**
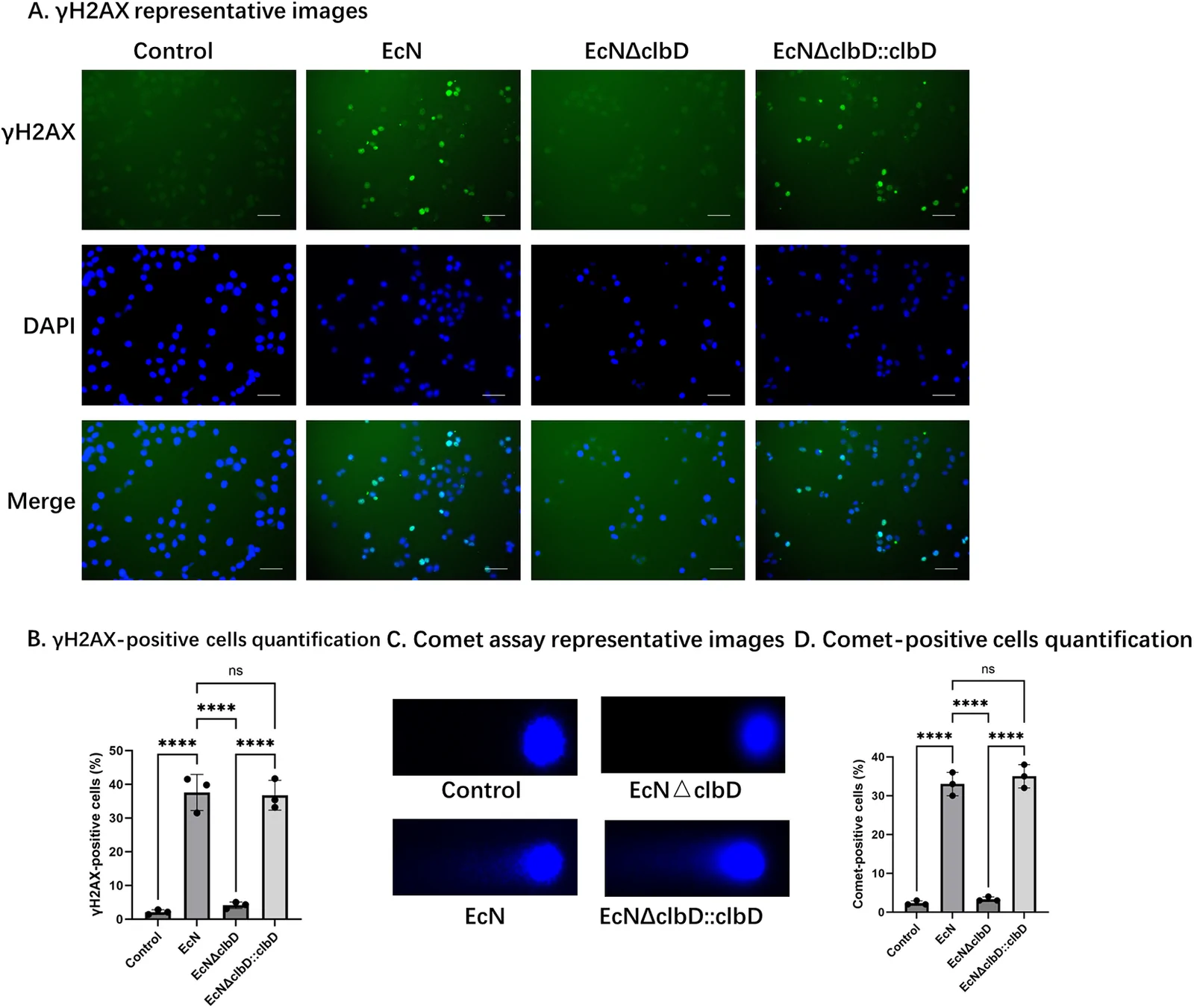
*clbD* deletion attenuates EcN-induced DNA damage responses, whereas chromosomal complementation restores the phenotype. HeLa cells were left untreated or co-cultured with EcN, EcNΔclbD, or EcNΔclbD::clbD for 4 h at an MOI of 10. **(A)** Representative immunofluorescence images showing γH2AX staining, DAPI-counterstained nuclei, and merged images. **(B)** Quantification of γH2AX-positive cells. **(C)** Representative alkaline comet assay images showing DNA migration after bacterial co-culture. **(D)** Quantification of comet-positive cells. For the comet assay, 100 cells were quantified per group in each independent experiment. Dots represent independent biological replicates, and bars indicate mean ± SEM. Statistical significance was determined by one-way ANOVA followed by Tukey’s multiple-comparisons test. ns, not significant; ****P < 0.0001. Scale bar, 200 μm for γH2AX immunofluorescence images.

To determine whether *clbD* deletion contributed to the reduction in EcN-induced DNA damage responses, we compared control cells, EcN-treated cells, EcNΔ*clbD*-treated cells, and EcNΔ*clbD*::*clbD*-treated cells. EcN markedly increased γH2AX positivity compared with the control group. In contrast, EcNΔ*clbD* significantly reduced γH2AX positivity to a level close to that of the control group. Importantly, chromosomal complementation of *clbD* restored the γH2AX response, with EcNΔ*clbD*::*clbD* inducing γH2AX positivity at a level comparable to EcN and significantly higher than EcNΔ*clbD* (**Figures 2A, B**).

To further validate DNA damage responses using an orthogonal assay, we performed alkaline comet assays after bacterial co-culture. Consistent with the γH2AX immunofluorescence results, EcN markedly increased the proportion of comet-positive cells, whereas EcNΔ*clbD* substantially reduced this proportion. Chromosomal complementation of *clbD* restored the comet-positive phenotype, with EcNΔ*clbD*::*clbD* showing a comet-positive rate comparable to that of EcN (**Figures 2C, D**). Together, these results indicate that *clbD* deletion attenuates EcN-induced DNA damage responses under the present co-culture conditions, and that restoration of *clbD* reverses this phenotype, supporting a causal role of *clbD* in EcN-induced DNA damage responses.

### EcNΔ*clbD* attenuates inflammatory gene induction and TLR4–MYD88–NFKB1-related transcriptional responses in NCM460 cells

**Figure 3:**
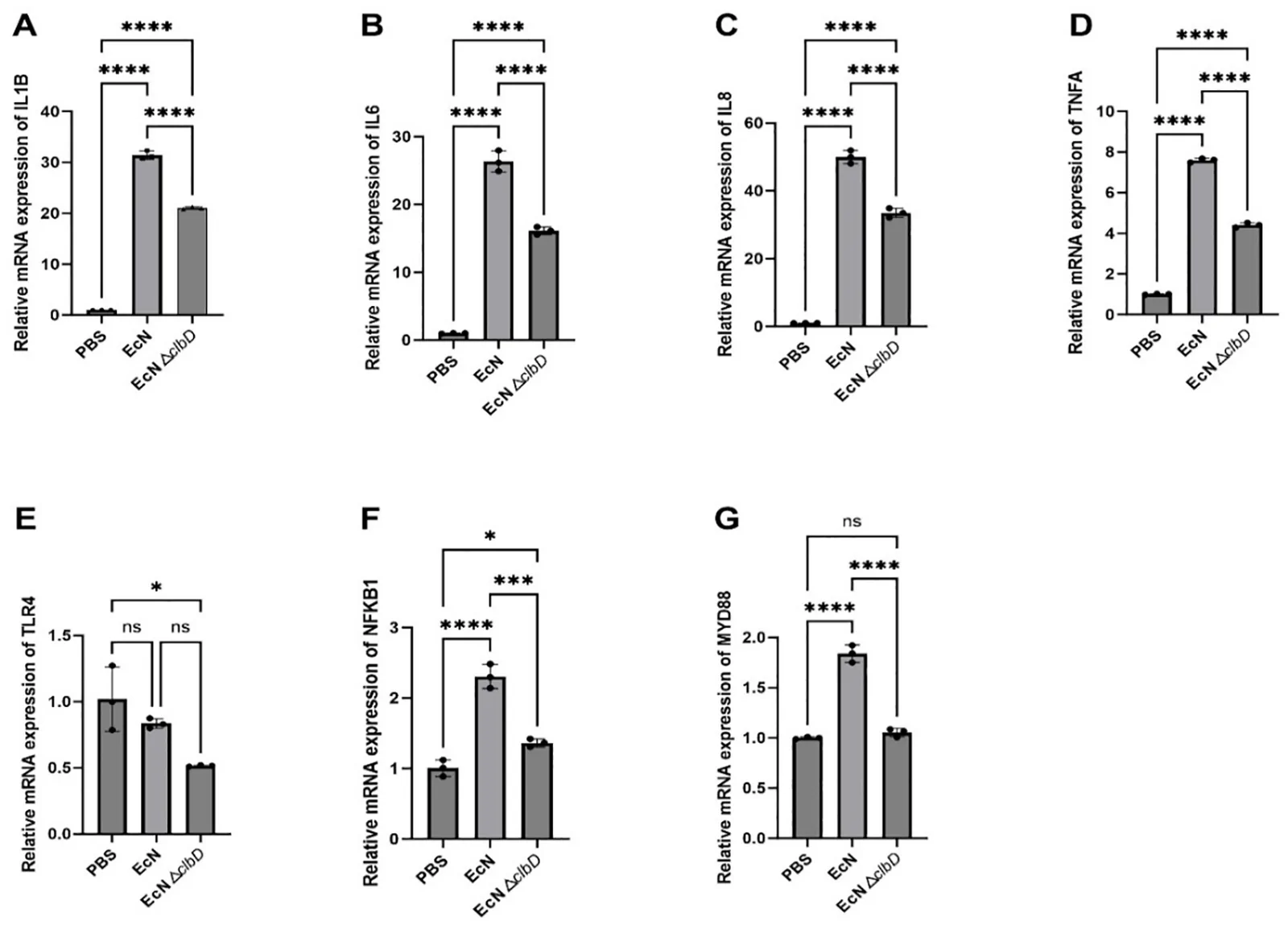
EcNΔclbD attenuates inflammatory gene induction and TLR4–MYD88–NFKB1-related transcriptional responses in NCM460 cells. NCM460 cells were co-cultured with PBS, EcN, or EcNΔclbD, followed by qPCR analysis of mRNA expression normalized to GAPDH. PBS indicates an equivalent volume of sterile PBS added as a mock control corresponding to bacteria washed twice with PBS before co-culture. **(A–D)** Relative mRNA expression of the pro-inflammatory cytokines IL1B, IL6, IL8, and TNFA. **(E–G)** Relative mRNA expression of TLR4, NFKB1, and MYD88. Data are shown as mean ± SEM; dots represent independent biological replicates. Statistical significance was determined by one-way ANOVA followed by Tukey’s multiple-comparisons test. ns, not significant; *P < 0.05; ***P < 0.001; ****P < 0.0001.

We next examined whether *clbD* deletion was associated with altered inflammatory transcriptional responses in NCM460 colonic epithelial cells after bacterial co-culture. Compared with PBS-treated control cells, EcN markedly increased the mRNA expression of pro-inflammatory cytokines, including IL1B, IL6, IL8, and TNFA. In contrast, EcNΔ*clbD* induced substantially lower expression of these inflammatory transcripts than EcN, although the expression levels of several cytokines remained higher than those in the PBS control group (**Figures 3A–D**).

We also assessed the mRNA expression of genes related to the TLR4–MYD88–NFKB1 axis. EcN increased NFKB1 and MYD88 mRNA expression compared with the PBS control, whereas EcNΔ*clbD* reduced this transcriptional induction. TLR4 expression showed a different pattern, with EcNΔ*clbD* showing lower TLR4 mRNA expression than the PBS control and EcN groups (**Figures 3E–G**). These results indicate that EcNΔ*clbD* elicits a weaker inflammatory transcriptional response than EcN in NCM460 cells under the present co-culture conditions. Because these analyses were performed at the mRNA level, they should be interpreted as transcriptional changes rather than direct evidence of pathway activation or protein-level inflammatory output.

To further evaluate potential confounding factors in the co-culture system, we performed additional control experiments comparing the two bacterial exposure conditions directly. First, to assess whether the reduced inflammatory transcription in the EcNΔ*clbD* group could be explained by differential host cell metabolic activity, we performed a CCK-8 assay after bacterial co-culture. After bacterial exposure, NCM460 cells were washed twice with PBS to remove non-adherent bacteria and then incubated in antibiotic-containing medium before CCK-8 measurement. No significant difference in CCK-8 absorbance at 450 nm was observed between EcN- and EcNΔ*clbD*-treated NCM460 cells (**Supplementary Figure 3**).

Second, to determine whether the weaker inflammatory transcription induced by EcNΔ*clbD* could be explained by reduced bacterial association with NCM460 cells, we quantified cell-associated bacterial loads after washing. NCM460 cells were washed twice with PBS, lysed, serially diluted, and plated on LB agar for CFU enumeration. No significant difference in cell-associated bacterial loads was observed between EcN and EcNΔ*clbD* (**Supplementary Figure 4**). Together, these control experiments suggest that the reduced inflammatory transcription observed in the EcNΔ*clbD* group was unlikely to be primarily explained by major differences in host cell CCK-8 readout or cell-associated bacterial burden between the two bacterial exposure conditions.

### *clbD* deletion does not cause detectable morphological changes in EcN

**Figure 4:**
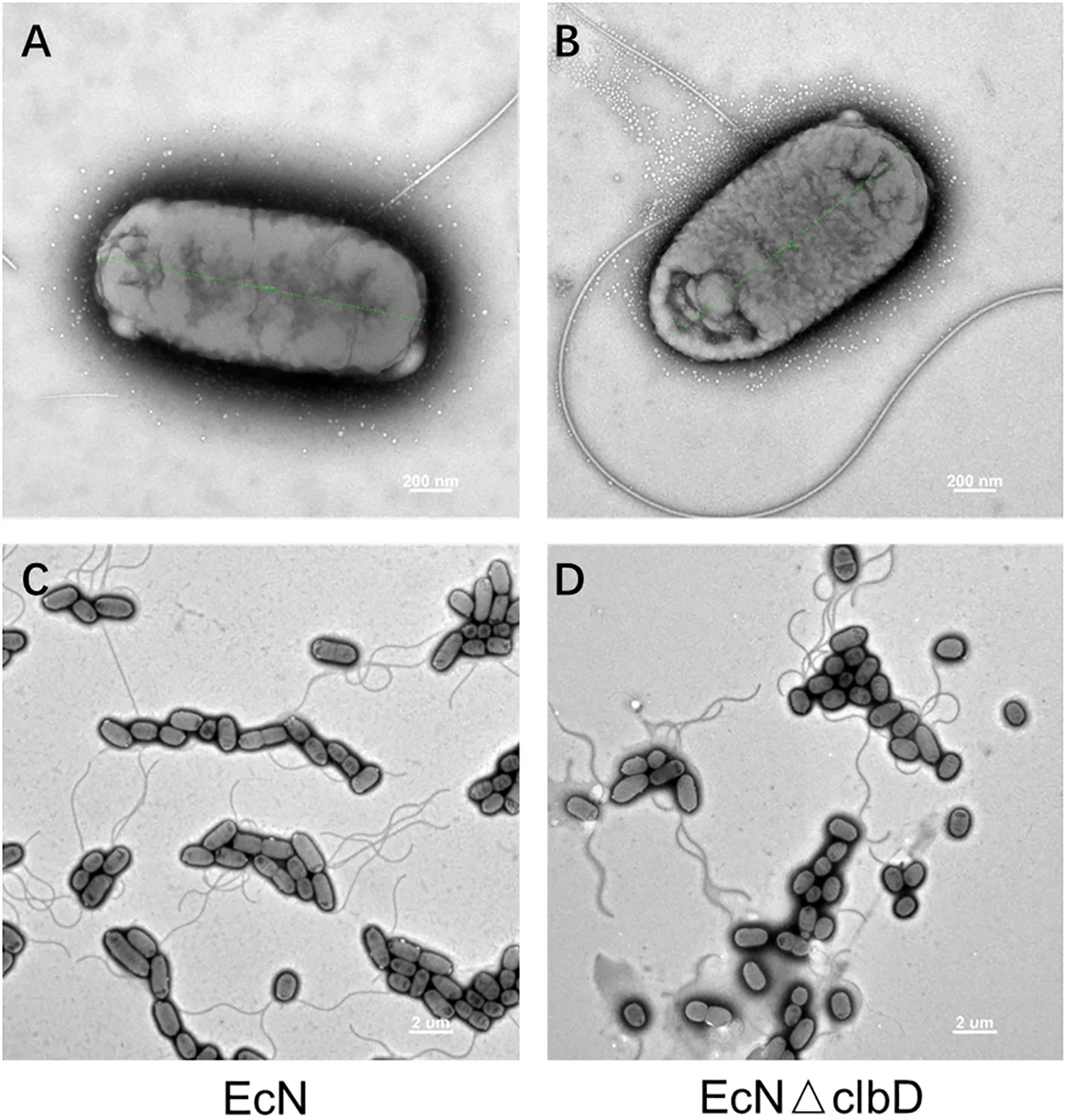
Deletion of *clbD* does not cause detectable morphological or ultrastructural alterations in EcN. Representative TEM images of EcN and EcNΔclbD. **(A, B)** High-magnification views (scale bar, 200 nm). **(C, D)** Low-magnification views showing overall morphology and distribution (scale bar, 2 μm).

To assess whether *clbD* deletion caused gross bacterial morphological alterations, we examined EcN and EcNΔ*clbD* by transmission electron microscopy. At high magnification, both strains displayed typical rod-shaped morphology with intact cell envelopes and no overt ultrastructural abnormalities (**Figures 4A, B**). At low magnification, the overall morphology and distribution patterns were comparable between EcN and EcNΔ*clbD* (**Figures 4C, D**). These observations suggest that *clbD* deletion did not cause detectable morphological or ultrastructural disruption under the TEM conditions used in this study.

### Untargeted metabolomics suggests exploratory metabolic shifts associated with *clbD* deletion

**Figure 5:**
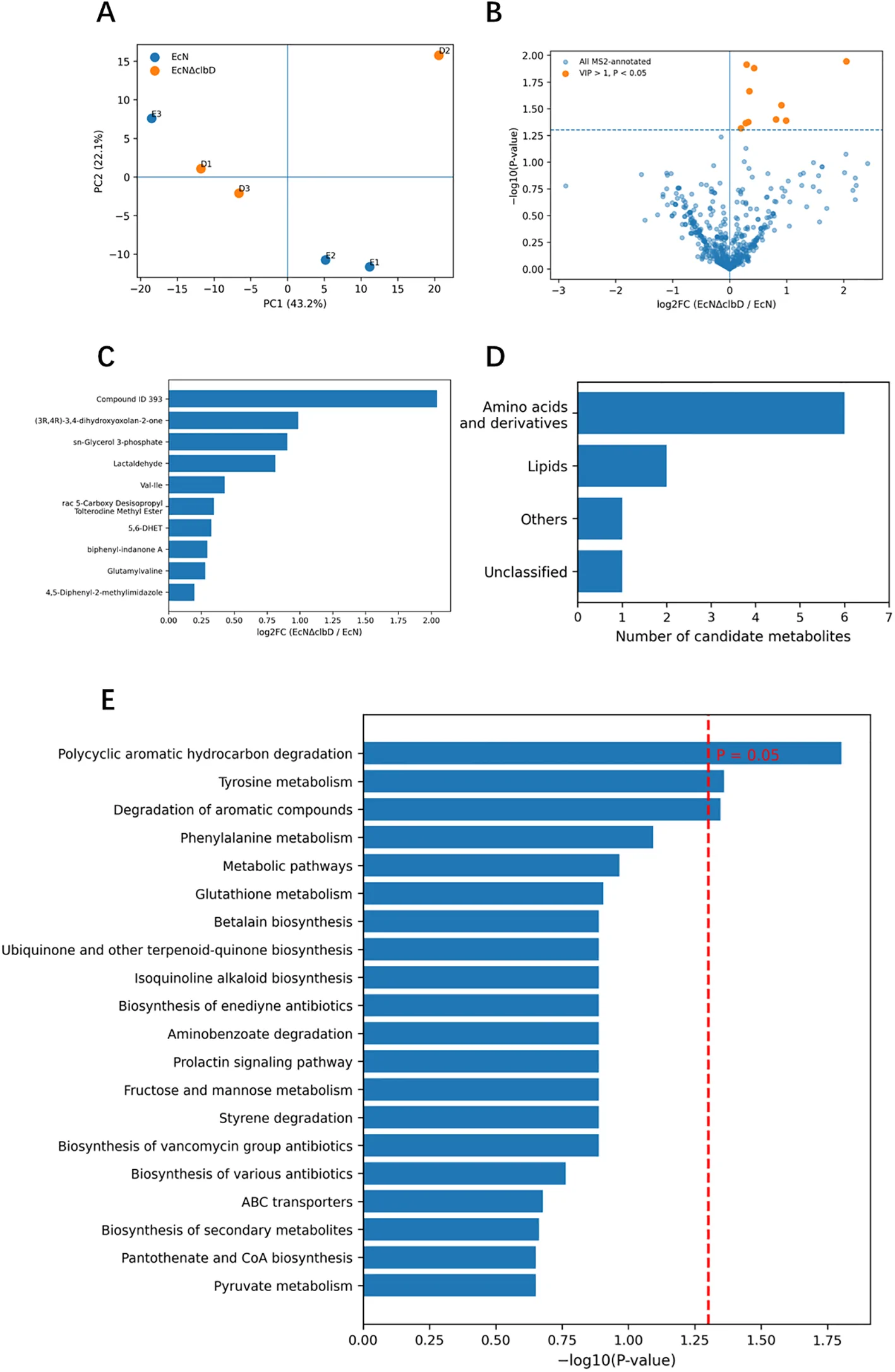
Untargeted metabolomics suggests exploratory metabolic shifts in EcNΔclbD relative to EcN. **(A)** PCA based on 509 MS2-annotated metabolites shows a separation trend between groups. **(B)** Volcano plot of differential features [log2FC, EcNΔclbD/EcN; −log10(P-value)]. Candidate metabolites were defined as VIP > 1 and P < 0.05, and the dashed line indicates P = 0.05. **(C)** Candidate metabolites highlighting the direction and magnitude of changes. Compound ID 393 represents a long-name annotated metabolite, with the full annotation provided in **Supplementary Table 3**. **(D)** Chemical superclass distribution of candidate metabolites. **(E)** KEGG pathway enrichment using candidates with KEGG compound IDs. The top 20 pathways are shown, and the red dashed line indicates P = 0.05.

To explore whether *clbD* deletion was associated with broader metabolic changes, we performed untargeted metabolomics comparing EcNΔ*clbD* with EcN using three biological replicates per group. PCA based on 509 MS2-annotated metabolites showed a separation trend between EcNΔ*clbD* and EcN, suggesting a possible metabolic shift associated with *clbD* deletion (**Figure 5A**). Given the limited sample size, these metabolomics data were interpreted as exploratory and hypothesis-generating rather than confirmatory.

For candidate screening, multivariate importance and univariate testing were combined. Candidate metabolites were defined as metabolites with VIP > 1 and P < 0.05. Fold changes were calculated and presented as log2FC (EcNΔ*clbD*/EcN). Under this criterion, 10 MS2-annotated candidate metabolites were identified (**Figure 5B**). These candidate metabolites showed higher abundance in EcNΔ*clbD* than in EcN and included sn-glycerol 3-phosphate, lactaldehyde, Val-Ile, 5,6-DHET, biphenyl-indanone A, glutamylvaline, and several additional annotated compounds (**Figure 5C**). Because no multiple-testing correction was applied and only three biological replicates were included per group, these metabolite-level differences should be interpreted cautiously.

The chemical superclass distribution of the candidate metabolites was further summarized. Amino acids and derivatives represented the largest category, followed by lipids, other compounds, and unclassified compounds (**Figure 5D**). Candidate metabolites with KEGG compound identifiers were then used for exploratory pathway enrichment, and the top enriched pathways are shown in **Figure 5E**. Several enriched pathways reached nominal P < 0.05, including polycyclic aromatic hydrocarbon degradation, tyrosine metabolism, and degradation of aromatic compounds. However, because of the small sample size, limited number of mapped candidate metabolites, incomplete pathway coverage, and unadjusted P values, these pathway-level results should not be interpreted as mechanistic evidence. Instead, they provide preliminary clues for future targeted metabolomic validation and functional follow-up studies. Full candidate metabolite lists, background metabolite annotations, and KEGG enrichment outputs are provided in **Supplementary Table 3**.

### EcNΔ*clbD* shows condition-dependent changes in gastrointestinal stress tolerance

**Figure 6:**
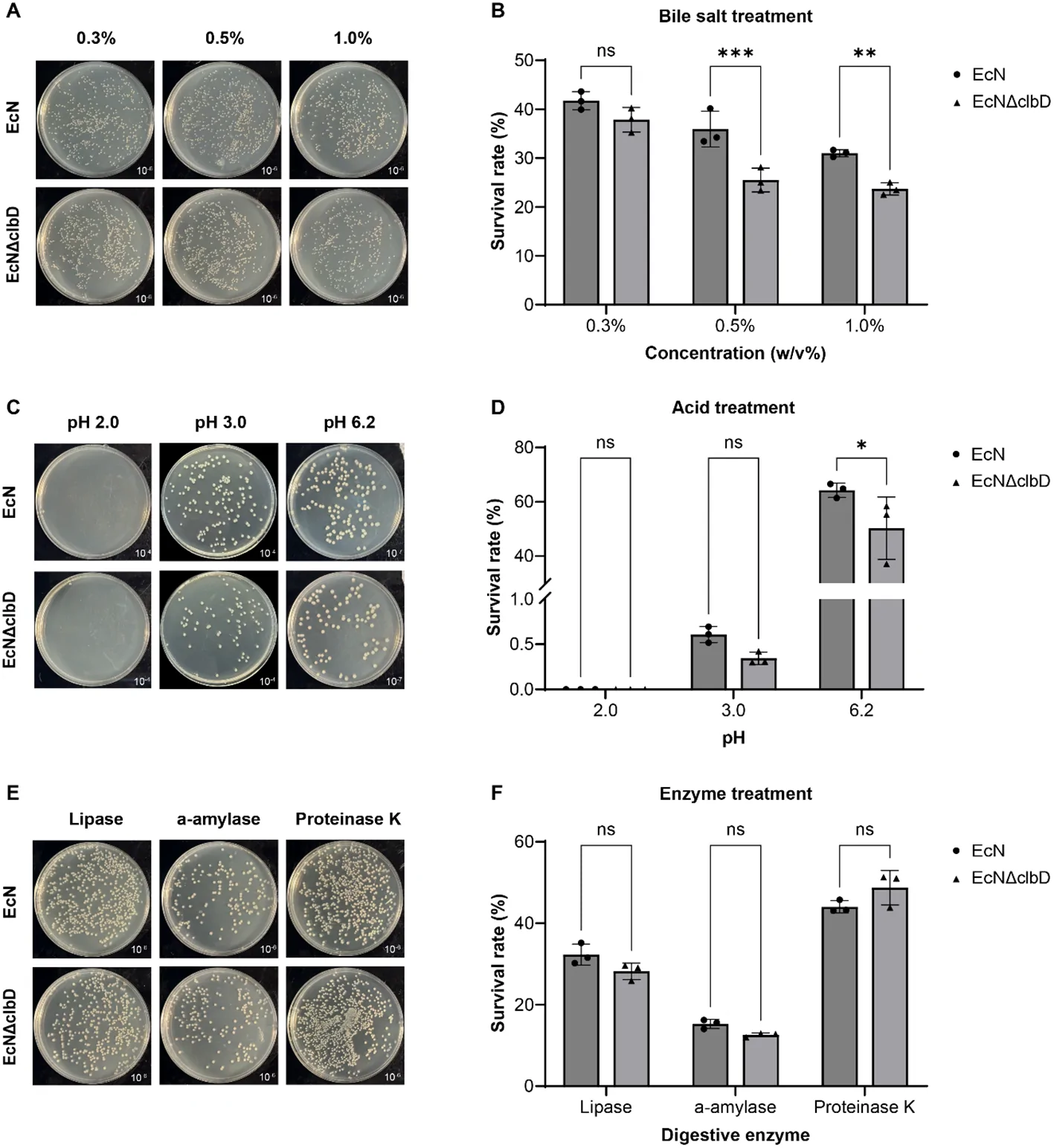
Survival of EcN and EcNΔclbD under gastrointestinal stress conditions. EcN and EcNΔclbD were exposed to bile salt, acid, or digestive enzyme treatments, and viable bacteria were quantified by serial dilution and plating. **(A)** Representative colony images after bile salt treatment at 0.3%, 0.5%, and 1.0%. **(B)** Quantification of bacterial survival after bile salt treatment. **(C)** Representative colony images after acid treatment at pH 2.0, pH 3.0, and pH 6.2. Representative plates are shown at different dilution factors to obtain countable colonies: pH 2.0 and pH 3.0 plates were imaged from 10⁻^4^ dilutions, whereas pH 6.2 plates were imaged from 10⁻^7^ dilutions. **(D)** Quantification of bacterial survival after acid treatment based on dilution-corrected CFU counts. **(E)** Representative colony images after digestive enzyme treatments with lipase, α-amylase, and proteinase K. **(F)** Quantification of bacterial survival after digestive enzyme treatment. Survival rates were calculated as 100 × CFU after treatment/CFU before treatment. Data are shown as mean ± SEM; dots represent independent biological replicates. Statistical significance was determined separately for each stress category using two-way ANOVA followed by Šídák’s multiple-comparisons test. ns, not significant; *P < 0.05; **P < 0.01; ***P < 0.001.

To evaluate whether *clbD* deletion affected bacterial robustness under gastrointestinal-relevant stress conditions, we compared the survival of EcN and EcNΔ*clbD* after bile salt, acid, and digestive enzyme treatments. Representative colony images and dilution-corrected survival rates are shown in **Figure 6**. To address multiple comparisons across stress conditions, each stress category was analyzed using two-way ANOVA followed by Šídák’s multiple-comparisons test.

Under bile salt exposure, EcNΔ*clbD* showed survival comparable to EcN at 0.3% bile salts, whereas its survival was significantly reduced at higher bile salt concentrations, including 0.5% and 1.0% (**Figures 6A, B**). Under acid treatment, both strains showed nearly undetectable survival at pH 2.0. At pH 3.0, survival remained very low in both groups, and no significant difference was observed between EcN and EcNΔ*clbD*. At pH 6.2, EcNΔ*clbD* showed a modest but significant reduction in survival compared with EcN (**Figures 6C, D**). Under digestive enzyme treatments, including lipase, α-amylase, and proteinase K, no significant differences in survival were observed between EcN and EcNΔ*clbD* (**Figures 6E, F**).

Together, these results indicate that *clbD* deletion did not cause a generalized loss of viability across all gastrointestinal stressors tested. Instead, EcNΔ*clbD* displayed condition-dependent changes in stress tolerance, most notably under higher bile salt exposure. To determine whether these stress-tolerance differences reflected a general growth defect, we compared baseline growth kinetics under standard LB culture conditions. EcNΔ*clbD* displayed a growth curve comparable to that of EcN, including similar exponential-phase growth and stationary-phase OD600 levels (**Supplementary Figure 5**). These data suggest that the stress-tolerance differences observed under selected gastrointestinal stress conditions were unlikely to result from a generalized growth impairment of EcNΔ*clbD*.

## Discussion

The increasing use of *Escherichia coli* Nissle 1917 (EcN) as both a conventional probiotic and an engineering chassis for live biotherapeutic applications has intensified the need for safety-by-design strain optimization. Although EcN has a long history of probiotic use, including evidence supporting maintenance of remission in ulcerative colitis (Kruis et al., 2004), its carriage of the *pks* genomic island remains a major safety concern. The *pks* island encodes the biosynthetic machinery for colibactin, and colibactin-producing *pks*-positive *Escherichia coli* has been associated with DNA damage, mutational signatures, and colorectal cancer-related risk in susceptible contexts (Nougayrède et al., 2006; Nougayrède et al., 2021). In this setting, targeted genetic attenuation of colibactin-associated risk represents an important strategy for developing safety-optimized EcN chassis strains.

In the present study, deletion of *clbD* markedly attenuated EcN-induced DNA damage responses. Compared with EcN, EcNΔ*clbD* substantially reduced γH2AX positivity and comet-positive cells, whereas chromosomal complementation of *clbD* restored both readouts to levels comparable to EcN. The inclusion of EcNΔ*clbD*::*clbD* strengthens the causal interpretation that the observed reduction in DNA damage response readouts is attributable to *clbD* disruption rather than nonspecific effects of genome editing or strain handling. Importantly, we interpret these results as attenuation of DNA damage responses rather than complete elimination of genotoxicity. γH2AX reflects DNA damage-associated signaling, and the alkaline comet assay provides an orthogonal measure of DNA strand break-related migration; together, these assays support reduced DNA damage responses under the present co-culture conditions, but they do not directly quantify colibactin production or fully exclude residual genotoxic activity.

The local transcriptional analysis of *pks* genes further refines the interpretation of the *clbD* deletion strategy. As expected, *clbD* expression was nearly undetectable in EcNΔ*clbD* and was restored in EcNΔ*clbD*::*clbD*. We also observed detectable changes in the neighboring *pks* genes *clbC* and *clbE* after *clbD* deletion, whereas chromosomal complementation restored the local transcriptional profile toward the parental EcN pattern. These findings indicate that *clbD* deletion may have local transcriptional effects within the *pks* island, highlighting the importance of assessing neighboring genes when interpreting phenotypes caused by deletion of a single *pks* gene. Nevertheless, the restoration of both local transcriptional patterns and DNA damage response phenotypes by *clbD* complementation supports the conclusion that *clbD* is functionally linked to EcN-induced DNA damage responses in this system.

In addition to DNA damage response readouts, EcNΔ*clbD* induced weaker inflammatory transcriptional responses in NCM460 colonic epithelial cells than EcN. Specifically, EcNΔ*clbD* reduced the induction of pro-inflammatory cytokine transcripts and TLR4–MYD88–NFKB1-related transcripts compared with EcN. These findings are consistent with the possibility that reduced epithelial stress and DNA damage responses may secondarily attenuate inflammatory transcription. However, alternative explanations remain possible, including broader effects of *pks*-associated metabolism, bacterial surface properties, or host–microbe interaction signals. Because our inflammatory analyses were performed at the mRNA level, they should not be interpreted as direct evidence of protein-level cytokine output or pathway activation. Future studies using cytokine ELISA, immunoblotting for pathway activation markers, and functional epithelial barrier assays will be needed to define the inflammatory consequences of *clbD* deletion more completely.

To address potential confounding factors in the epithelial co-culture system, we added host cell CCK-8 and cell-associated bacterial load controls. No significant difference in CCK-8 absorbance was observed between EcN- and EcNΔ*clbD*-treated NCM460 cells, suggesting that the reduced inflammatory transcription was unlikely to be primarily explained by a major difference in this host cell metabolic activity readout. Similarly, cell-associated bacterial loads after washing were comparable between EcN and EcNΔ*clbD*, indicating that the weaker inflammatory transcriptional response was not readily attributable to reduced bacterial association or exposure. These controls strengthen the interpretation of the inflammatory transcription data, although they do not replace direct protein-level or mechanistic validation.

A key translational question is whether *pks* attenuation alters bacterial traits relevant to probiotic or chassis performance. Previous studies indicate that the relationship between *pks* disruption and probiotic-relevant function is context-dependent. Some genetic strategies targeting the colibactin pathway can reduce genotoxicity-associated readouts while preserving selected bacterial functions, whereas other approaches may affect competitive fitness, host interaction, or stress-related phenotypes (Massip et al., 2019; Kalantari et al., 2023). Our gastrointestinal stress assays support this broader view. EcNΔ*clbD* did not show a generalized loss of viability or a baseline growth defect in LB medium. Instead, reduced survival was observed under selected stress conditions, most notably higher bile salt exposure and a modest reduction at pH 6.2, whereas no significant survival differences were observed under digestive enzyme treatments. Thus, *clbD* deletion appears to produce condition-dependent fitness effects rather than a global growth impairment. This distinction is important because future applications of EcNΔ*clbD* may require formulation, encapsulation, dosing optimization, or additional engineering depending on the intended route and therapeutic context (Lynch et al., 2022).

Transmission electron microscopy showed no detectable morphological or ultrastructural disruption in EcNΔ*clbD* compared with EcN, suggesting that the observed host-response and stress-tolerance differences are unlikely to result from gross bacterial structural abnormalities. However, TEM cannot exclude more subtle alterations in membrane composition, outer membrane vesicle production, surface-associated molecules, or metabolic state. EcN-derived outer membrane vesicles have been reported to modulate innate immune responses and barrier-related phenotypes, supporting the possibility that bacterial surface and vesicle-associated factors may influence epithelial signaling (Hu et al., 2020; Han et al., 2024). In this context, our untargeted metabolomics analysis suggested an exploratory metabolic shift associated with *clbD* deletion. Because the metabolomics experiment included only three biological replicates per group and did not apply multiple-testing correction, these results should be considered hypothesis-generating rather than mechanistic proof. Targeted metabolomics and functional rescue experiments will be required to determine whether specific metabolic changes contribute to altered stress tolerance or host responses.

The safety optimization of EcN should also be considered in light of recent studies showing that colibactin-associated risk depends not only on toxin biosynthesis but also on host–microbe contact determinants. For example, adhesin-mediated epithelial binding has been implicated in colibactin-driven colorectal cancer models, suggesting that bacterial adhesion, colonization, and epithelial proximity can modulate genotoxic outcomes (Jans et al., 2024; Ouyang and Puschhof, 2024). In parallel, chemical biology studies have shown that pharmacological targeting of the colibactin-associated genotoxic pathway can reduce colibactin-related DNA damage, further supporting this pathway as an actionable safety target (Volpe et al., 2023). These findings collectively support a multi-layer safety framework in which colibactin biosynthesis, toxin activation, bacterial adhesion, genetic stability, and host-context dependence are all considered during EcN chassis development.

Several limitations should be emphasized. First, although γH2AX immunofluorescence and comet assay provide complementary DNA damage response readouts, we did not directly measure colibactin or colibactin-derived DNA adducts. Therefore, the present data support reduced DNA damage responses rather than direct biochemical proof that colibactin biosynthesis or activity was completely abolished. Second, host-response experiments were performed in limited *in vitro* cell systems. Inflammatory transcription was assessed in NCM460 cells, a non-tumorigenic colonic epithelial cell model selected to reflect a non-cancerous epithelial context. Nevertheless, responses to EcN and EcNΔ*clbD* may vary across epithelial models. Future studies should validate these findings in additional non-tumorigenic intestinal epithelial cells, intestinal organoids, and, where appropriate, colorectal cancer-derived epithelial models. Third, this study did not directly evaluate canonical probiotic functions, such as pathogen antagonism, mucosal colonization, barrier protection, or disease-model efficacy. Therefore, the current findings should not be generalized to overall probiotic performance or clinical safety.

Future validation in inflammatory bowel disease-relevant models will be important. EcN-derived bacterial ghosts have been reported to alleviate inflammatory bowel disease phenotypes in zebrafish, supporting the utility of EcN-derived systems in intestinal inflammatory models (Chen et al., 2023b). More recent engineered nanoplatforms using bacterial ghost-based targeting strategies also highlight the potential of combining anti-inflammatory and microbiota-modulating actions for chronic inflammatory bowel disease therapy (Fang et al., 2026). These studies provide useful context for future work, but they should not be interpreted as direct evidence for the efficacy of EcNΔ*clbD*. Instead, they support the need to evaluate safety-optimized EcN derivatives in disease-relevant *in vivo* systems, including models that assess inflammation, barrier function, microbiota interactions, persistence, and long-term safety.

In conclusion, chromosomal *clbD* deletion attenuated EcN-induced DNA damage responses and epithelial inflammatory transcriptional responses *in vitro*, while *clbD* complementation restored the DNA damage response phenotype. *clbD* deletion did not cause detectable ultrastructural disruption or a generalized growth defect, but it was associated with condition-dependent changes in gastrointestinal stress tolerance and exploratory metabolic shifts. Together, these findings support EcNΔ*clbD* as a candidate safety-optimized EcN chassis that requires further validation in additional epithelial models, disease-relevant *in vivo* systems, and probiotic function assays before broader translational conclusions can be drawn.

## Data Availability

The original contributions presented in the study are included in the article/**Supplementary Material**. Further inquiries can be directed to the corresponding author.
