# Supplementary material for: Towards a safer probiotic: clbD deletion attenuates EcN-induced DNA damage responses and epithelial inflammatory transcriptional responses
Source: Front Cell Infect Microbiol. 2026 Aug 6;16:1812973. doi: 10.3389/fcimb.2026.1812973 (PMC13489827; doi:10.3389/fcimb.2026.1812973)
Supplement: Supplementary file 1 [file DataSheet1.docx]

Supplementary Fig. S1.

Supplementary Figure S1. Uncropped gel images for PCR validation of clbD deletion and chromosomal complementation.

(A) Full, uncropped agarose gel image showing PCR products amplified from EcN, EcNΔclbD, and EcNΔclbD::clbD. The dashed red box indicates the region shown in panel B. (B) Cropped gel image corresponding to the boxed region in panel A, highlighting the expected PCR amplicons. EcN produced the expected 1561-bp amplicon, EcNΔclbD produced a shorter 691-bp amplicon consistent with clbD deletion, and EcNΔclbD::clbD restored the 1561-bp amplicon. M, DNA marker.

Supplementary Fig. S2.

Supplementary Figure S2. Transcriptional assessment of clbD and neighboring pks genes.

Relative mRNA expression of clbC, clbD, and clbE was measured by qPCR in EcN, EcNΔclbD, and EcNΔclbD::clbD. Expression levels were normalized to the internal reference gene and are shown relative to EcN. Dots represent independent biological replicates, and bars indicate mean ± SEM. Statistical significance was determined by one-way ANOVA followed by Tukey’s multiple-comparisons test. ns, not significant; *P < 0.05; **P < 0.01; ***P < 0.001.

Supplementary Fig. S3.

Supplementary Figure S3. CCK-8 assessment of NCM460 cell metabolic activity after bacterial co-culture.

NCM460 cells were co-cultured with EcN or EcNΔclbD. After bacterial exposure, cells were washed twice with PBS to remove non-adherent bacteria and then incubated in antibiotic-containing medium before CCK-8 measurement. CCK-8 absorbance was measured at 450 nm. No significant difference was observed between EcN- and EcNΔclbD-treated cells. Data are shown as mean ± SEM; dots represent independent biological replicates. Statistical significance was determined by unpaired two-tailed Student’s t-test. ns, not significant.

Supplementary Fig. S4.

Supplementary Figure S4. Cell-associated bacterial loads after co-culture with NCM460 cells.

NCM460 cells were co-cultured with EcN or EcNΔclbD. After bacterial exposure, cells were washed twice with PBS to remove non-adherent bacteria, lysed, serially diluted, and plated on LB agar for CFU enumeration. Cell-associated bacterial loads were calculated as dilution-corrected CFU/mL. No significant difference was observed between EcN and EcNΔclbD. Data are shown as mean ± SEM; dots represent independent biological replicates. Statistical significance was determined by unpaired two-tailed Student’s t-test. ns, not significant.

Supplementary Fig. S5.

Supplementary Figure S5. Baseline growth characteristics of EcN and EcNΔclbD in LB medium.

Growth curves of EcN and EcNΔclbD were measured in LB medium by monitoring OD600 values over time. EcNΔclbD showed growth kinetics comparable to EcN under standard culture conditions, suggesting that clbD deletion did not cause an overt general growth defect in LB medium.
